# Effect of hypoxia on hypoxia inducible factor-1α, insulin-like growth factor I and vascular endothelial growth factor expression in hepatocellular carcinoma HepG2 cells

**DOI:** 10.3892/ol.2015.2879

**Published:** 2015-01-15

**Authors:** QIANG LIU, ZHENG XU, SHUNBAO MAO, WENYOU CHEN, RONGYAO ZENG, SONG ZHOU, JING LIU

**Affiliations:** Department of General Surgery, The 175th Hospital of PLA (Affiliated Dongnan Hospital of Xiamen University), Zhangzhou, Fujian 363000, P.R. China

**Keywords:** hepatocellular carcinoma, hypoxia, hypoxia inducible factor-1α, insulin-like growth factor I, vascular endothelial growth factor

## Abstract

Hypoxic microenvironments and angiogenesis have been a focus of tumor research in previous years. The aim of the the present study was to create a hypoxic model and observe the effect of hypoxia on the expression of hypoxia inducible factor-1α (HIF-1α), insulin-like growth factor I (IGF-1) and vascular endothelial growth factor expression. The hypoxia model was generated using cobalt chloride (CoCl_2_) and an MTT assay was used to observe the influence of hypoxia on HepG2 cells. Reverse transcription-polymerase chain reaction, western blotting, ELISA and confocal immunofluorescence microscopy were used to detect the expression of HIF-1α, IGF-1 and VEGF in HepG2 cells, in which hypoxia was induced by various concentrations of CoCl_2_ and for various incubation times. The cell viability worsened with increasing concentrations of CoCl_2_. The expression of HIF-1α and IGF-1R was observed in hypoxic HepG2 cells, with the exception of HIF-1α mRNA. The expression of IGF-1R and VEGF mRNA and protein was correlated with the concentration of CoCl_2_ and the time that hypoxia was induced for. The expression of HIF-1α mRNA and protein was positively correlated with the expression of the VEGF mRNA and protein in a dose- and time-dependent manner under hypoxic conditions. Using immunofluorescence, it was observed that IGF-1R and HIF-1α were secreted from the hypoxic HepG2 cells. It was concluded that hypoxia induces the accumulation of IGF-1R and HIF-1α mRNA and protein, which regulates the expression of VEGF mRNA and protein in hypoxic HepG2 cells.

## Introduction

Hepatocellular carcinoma (HCC) is the fifth most common cause of cancer and is also the third leading cause of cancer-associated mortality worldwide, with ~700,000 mortalities reported annually (1,2). The incidence of HCC has demonstrated a notable increase worldwide, particularly in developing countries within Asia and sub-Saharan Africa, where hepatitis B and C viral infections are endemic, and in regions where food contaminated with Aflatoxin B1 is consumed (3). HCC is usually diagnosed at an advanced, incurable and metastatic stage due to the usual absence of specific symptoms and history of cirrhosis. Thus, the five-year overall survival rate is <10%; the effects of surgical and chemo-radiation therapies are poor for advanced and metastatic stages of HCC (4).

HCC is a solid tumor with numerous blood vessels, and the most important characteristic of HCC is the uncontrollable cell proliferation, which leads to a novel hypoxic microenvironment with increased oxygen consumption. Therefore, it is important to establish a vascular and nutrient supply system to meet the oxygen demands (5,6). Hypoxia is one of the fundamental biological phenomena that are strongly associated with the development and aggressiveness of a wide variety of solid tumors, including HCC. It has been reported that hypoxia inducible factor-1 (HIF-1α), the key to mediating hypoxia-responsive genes, and insulin-like growth factor I (IGF-1), which is secreted in the liver, may potentially be synergistic in regulating the vascular endothelial growth factor (VEGF) expression in type 2 diabetes (7). The present study focused on whether VEGF expression in HCC is also regulated by HIF-1α and IGF-1.

In the current study, a model of hypoxia was created using cobalt chloride (CoCl_2_) and an MTT assay was used to observe the influence of hypoxia on the proliferation of HepG2 cells. The effect of hypoxia on HepG2 cell invasion and angiogenesis was examined, as well as the expression and correlation of HIF-1α, IGF-1 and VEGF under hypoxic conditions.

## Materials and methods

### Drug and reagents

Dulbecco’s modified Eagle’s medium (DMEM), CoCl_2_ dissolved in DMEM, 3-(4,5-dimethylthiazol-2-yl)-2,5-diphenyltetrazolium bromide (MTT) assay kit, RIPA buffer, goat anti-rabbit fluorescein isothiocyanate immunoglobulin (Ig)G and tetramethylrhodamine isothiocyanate IgG antibodies were purchased from Sigma-Aldrich (St Louis, MO, USA). Goat anti-rabbit HIF-1α, IGF-1R and VEGF monoclonal antibodies were purchased from Cell Signaling Technology (Beverly, MA, USA). Goat anti-rabbit horseradish peroxidase (HRP) conjugated secondary and β-actin monoclonal antibodies were purchased from Santa Cruz Biotechnology, Inc. (Dallas, TX, USA). TRIzol^®^ reagent was purchased from Invitrogen (Carlsbad, CA, USA). Revertaid First Strand cDNA Synthesis Kit was purchased from Thermo Fisher Scientific (Waltham, MA, USA).

### Cell culture

The human hepatocellular carcinoma cell HepG2 was obtained from the American Type Culture Collection. HepG2 cells were maintained in DMEM medium supplemented with 10% fetal bovine serum (FBS), 100 units/ml penicillin and 100 μg/ml streptomycin. The HepG2 cells were maintained under the standard culture and normoxic conditions of 21% O_2_ and 5% CO_2_, at 37°C.

### Establishing the hypoxia model and groups

The HepG2 cells were plated in six-well plates in medium containing 10% FCS and grown to 70–80% confluency. Subsequently, the cells were washed extensively with serum-free DMEM medium and incubated with CoCl_2_ DMEM medium. The cells were divided into two groups. Since the results from the concentration-based experiment would affect the concentration used in the time-based experiment, according to previous studies and our preliminary experiment, the first group was treated with 0, 50, 100, 200, 400, 800 or 2,000 μmol/l CoCl_2_, and the cells were then maintained under standard culture conditions for 12 h (8–10). The second group was treated with 200 μmol/l CoCl_2_, but was incubated for 0, 4, 8, 12, 24 or 48 h.

### Cell survival assay

An MTT assay was conducted to investigate the effect of the growth and proliferation of HepG2 cells under CoCl_2_-induced hypoxic conditions. Overall, 5,000 cells were seeded into each well of a 96-well plate and were incubated under normoxic conditions overnight. The cells were then treated with 0, 50, 100, 200, 400, 800 or 2,000 μmol/l CoCl_2_ and incubated under standard culture conditions for 12 h. Cell proliferation was determined using an MTT assay (Sigma-Aldrich) according to the manufacturer’s instructions. The optical density (OD) was determined at 570 nm on SpectraMax M2 (Molecular Devices LLC, Sunnyvale, CA, USA). The experiment was repeated in triplicate.

### Quantitative PCR analysis to assess the mRNA levels of HIF-1α, IGF-1R and VEGF

Total RNA was isolated from cells using TRIzol^®^ Reagent. Quantitative PCR analysis of HIF-1α, IGF-1R and VEGF mRNA levels was performed using the One-step RT-PCR kit from Thermo Fisher Scientific, according to the manufacturer’s instructions. The following primers were designed for quantitative PCR: HIF-1α forward, 5′-ACTAAAGGACAAGTCACCACAGGA-3′ and reverse, 5′-TGCTGAATAATACCACTCACAACG-3′; IGF-1R forward, 5′-CTCAGTTAATCGTGAAGTGGAACC-3′ and reverse 5′-GCAGTAATTGTGCCGGTAAAGG-3′; VEGF forward, 5′-GAGGGCAGAATCATCACGAAGT-3′ and reverse, 5′-TCCTATGTGCTGGCCTTGGTGA-3′; and β-actin forward, 5′-CACACAGGAGAGGTGATAGCAAGT-3′ and reverse, 5′-GACCAAAAGCCTTCATACATCTCA-3′. All primers were synthesized by Shanghai Sangon Biological Engineering Technology and Services Co., Ltd. (Shanghai, China). The thermocycling conditions were as follows: 95°C for 5 min; 72°C for 10 min; and 40 cycles at 95°C for 10 sec and 50°C for 30 sec for HIF-1α and IGF-1R, or 60°C for 30 sec for VEGF. The relative HIF-1α, IGF-1R and VEGF mRNA levels were normalized to β-actin. The experiment was repeated in triplicate.

### Western blot analysis to assess the protein levels of HIF-1α, IGF-1R and VEGF

Total protein was isolated from cells using RIPA buffer reagent. The cells were then incubated at 4°C for 1 h. The lysates were ultrasonicated and centrifuged at 12,000 × g for 10 min. The supernatants were collected and stored at −70°C. Protein concentrations were determined using the bicinchoninic acid method. Protein (50–100 μg) was separated on a 10% polyacrylamide-SDS gel and electroblotted onto a nitrocellulose membrane. Subsequent to being blocked with Tris-buffered saline/5% non-fat dry milk for 2 h, the membranes were incubated overnight at 4°C with antibodies against HIF-1α (cat. no. 3176; dilution, 1:1,000), IGF-1R (cat. no. 9750; dilution, 1:1,000) and VEGF (cat. no. 2463; dilution, 1:1,000) (all rabbit polyclonal IgG; Cell Signaling Technology), followed by incubation with a horseradish peroxidase-conjugated secondary antibody (cat. no. sc-2048; dilution, 1:1,000; rabbit polyclonal IgG; Santa Cruz Biotechnology, Inc., Dallas, TX, USA) for 45 min at room temperature. The signals were visualized by the enhanced chemiluminescence detection system [Life Science (Research, Education, Process Separations, Food Science), Bio-Rad Laboratories (Shanghai) Co., Ltd., Shanghai, China]. As a loading control, the blots were reprobed using a specific antibody against human β-actin (cat. no. sc-7210; dilution, 1:4,000; rabbit polyclonal IgG provided at 200 μg/ml; Santa Cruz Biotechnology, Inc.).

### ELISA assay to assess the production of VEGF

The concentration of VEGF protein in the conditioned medium subsequnet to treatment with the various crocetin concentrations and treatment times was determined using the human VEGF ELISA development kit (R&D Systems, Inc., Minneapolis, MN, USA), according to the manufacturer’s instructions. The results were normalized to the cell number (2×10^5^ cells/well) and the experiment was repeated in triplicate.

### Confocal immunofluorescence microscopy assay

The cells were plated onto glass culture slides coated with fibronectin or bovine serum albumin (BSA) and processed by immunofluorescence. The cells were fixed, permeabilized and blocked with BSA. The cells were incubated with HIF-1α and IGF-1R antibodies overnight at 4°C, washed and were incubated with fluorochrome-conjugated secondary antibodies. The cells were incubated with rhodamine phalloidin to stain the stress fibers. The nuclei were visualized by staining with Hoechst 33258. Images were captured using fluorescence confocal microscopy [VF1000, Olympus (China) Co., Ltd., Beijing, China].

### Statistical analysis

The data are presented as the mean ± standard deviation for three separate experiments. One-way analysis of variance and the Bonferroni correction were employed for statistical analysis using SPSS 13.0 software for Windows (SPSS, Inc., Chicago, IL, USA). Pearson analysis was used for correlation analysis. P<0.05 was considered to indicate a statistically significant difference.

## Results

### The effect of hypoxia on HepG2 cells

**Figure 1:**
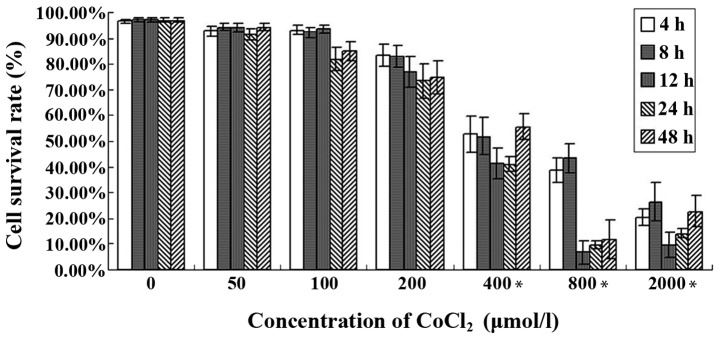
Effect of the CoCl_2_-induced hypoxia by on HepG2 cell viability was determined by an MTT assay. The HepG2 cells were treated with 0, 50, 100, 200, 400, 800 or 2,000 μmol/l CoCl_2_ and incubated at the standard culture conditions for 0, 4, 8, 12, 24 or 48 h. The cell survival rate (%) was correlated with the dose and time. *P<0.05 compared with control group (0 μmol/l). CoCl_2_, cobalt chloride.

In order to investigate the effect of hypoxia on HepG2 cells, an MTT assay was conducted using the human HepG2 cells. The results revealed that there was no significant difference in cell survival between the cells treated with 50, 100 and 200 μmol/l of CoCl_2_. However, when the cells were treated with a higher concentration or were incubated for a longer time, there was a significant difference between the cells treated with 400 μmol/l and those treated with 2,000 μmol/l CoCl_2_, and there was also a significant difference between the cells treated with 0 μmol/l and those treated with 800 μmol CoCl_2_ at the same incubation times (Fig. 1).

### Effect of CoCl_2_-induced hypoxia on HIF-1α, IGF-1R and VEGF mRNA expression

**Figure 2:**
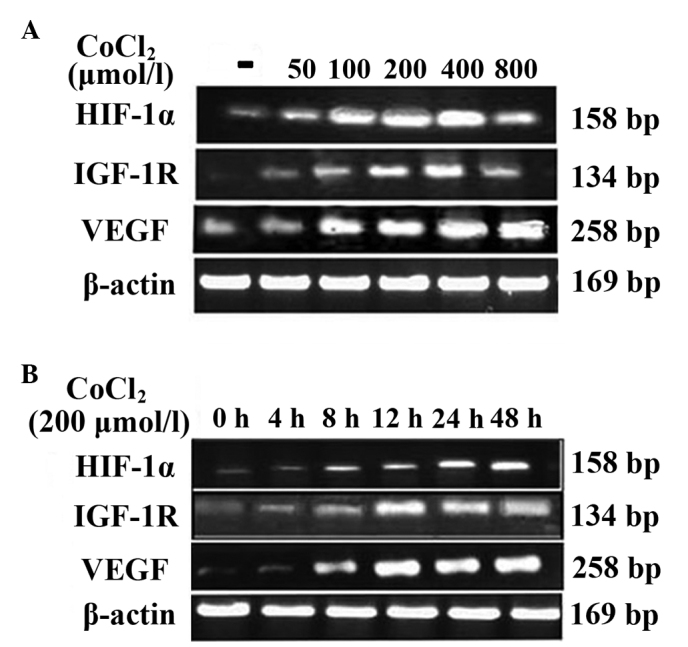
Effect of CoCl_2_- induced hypoxia on the expression of HIF-1α, IGF-1R and VEGF mRNA. (A) The HepG2 cells were treated with 0, 50, 100, 200, 400 or 800 μmol/l CoCl_2_ for 12 h. (B) The HepG2 cells were treated for 0, 4, 8, 12, 24 or 48 h with 200 μmol/l CoCl_2_. A significant difference in HIF-1α, IGF-1R and VEGF protein expression was observed when cells were treated with 200 and 400 μmol/l CoCl_2_, and for 12, 24 and 48 h, compared with the control (P<0.05). CoCl_2_, cobalt chloride; HIF-1α, hypoxia inducible factor-1α; ICF-1R, insulin-like growth factor-1 receptor; VEGF, vascular endothelial growth factor.

The quantitative RT-PCR assay was conducted to examine the effect of CoCl_2_-induced hypoxia on HIF-1α, IGF-1R and VEGF mRNA expression in human HepG2 cells. The cells were treated with 0, 50, 100, 200, 400 or 800 μmol/l of CoCl_2_ for 12 h. As shown in Fig. 2A, there was no apparent change in the HIF-1α mRNA levels in HepG2 cells. A significant difference in the expression of IGF-1R and VEGF mRNA between the cells treated with 200 and 400 μmol/l CoCl_2_ and the control group (Table I). The expression of HIF-1α mRNA was positively correlated with the expression of VEGF mRNA (r=0. 77; P<0.05) in a dose-dependent manner under hypoxic conditions.

The cells were also treated for 0, 4, 8, 12, 24 or 48 h with 200 μmol/l CoCl_2_. As shown in Fig. 2B, The expression of HIF-1α, IGF-1R and VEGF mRNA in the cells treated for 4 and 8 h was not significantly different compared with the expression in the control group, while there was a significant difference between the cells treated for 12, 24 and 48 h and the control group (Table II). It was also found that the expression of HIF-1α mRNA was positively correlated with the expression of VEGF mRNA (r=0.85, P<0.05), dependent on the incubtation time with CoCl_2_.

### Effect of CoCl_2_-induced hypoxia on HIF-1α, IGF-1R and VEGF protein expression

**Figure 3:**
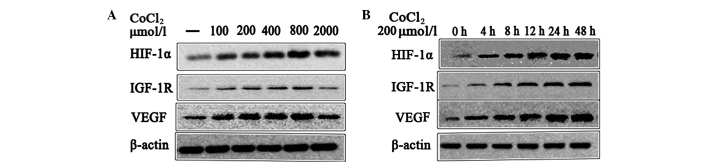
Effect of CoCl_2_-induced hypoxia on HIF-1α, IGF-1R and VEGF protein expression. (A) The HepG2 cells were treated with 0, 50, 100, 200, 400 or 800 μmol/l CoCl_2_ for 12 h. (B) The HepG2 cells were treated for 0,4,8,12, 24 or 48 h with 200 μmol/l CoCl_2_. A significant difference in HIF-1α, IGF-1R and VEGF mRNA expression was observed when cells were treated with 200 and 400 μmol/l CoCl_2_, and for 8, 12, 24 and 48 h, compared with the control (P<0.05). CoCl_2_, cobalt chloride; HIF-1α, hypoxia inducible factor-1α; ICF-1R, insulin-like growth factor-1 receptor; VEGF, vascular endothelial growth factor.

Western blot analysis was performed to examine the effect of CoCl_2_-induced hypoxia on HIF-1α, IGF-1R and VEGF protein expression in human HepG2 cells. The cells were treated with 0, 50, 100, 200, 400 or 800 μmol/l CoCl_2_ for 12 h. As shown in Fig. 3A, the expression of the HIF-1α, IGF-1R and VEGF proteins was not significantly different in the cells treated with 50, 100 and 800 μmol/l CoCl_2_ compared with the control group. However, the differences between the cells treated with 200 and 400 μmol/l and the control group were significantly different (Table III). In addition, it was also found that, under hypoxic conditions, the expression of the HIF-1α protein was positively correlated with the expression of the VEGF protein (r=0.90, P<0. 05) in a dose-dependent manner.

The cells were incubated for 0, 4, 8, 12, 24 or 48 h with 800 μmol/l CoCl_2_. As shown in Fig. 3B, there was no significant difference between the cells incubated for 4 h and the control group, whereas the cells incubated for 8, 12, 24 and 48 h were significantly different (Table IV). It was also found that, under hypoxic conditions, the expression of the HIF-1α protein was positively correlated with expression of the VEGF protein (r=0.78, P<0.05) in a time-dependent manner.

### Effect of HIF-1α and IGF-1 expression in hypoxic HepG2 cells

**Figure 4:**
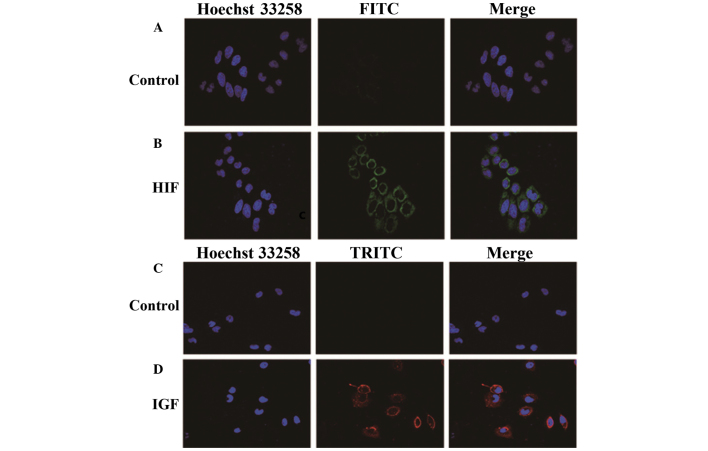
Confocal immunofluorescence microscopy of hypoxic HepG2 cells. The hypoxic HepG2 cells were seeded onto coverslips and stained with either control FITC (green) or TRITC (red). The nuclei were visualized using hoechst 33258 (blue). A, B, C and D represent the normoxia control group, HIF group, normoxia control group and IGF group, respectively. The Merge column shows that HIF-1 and IGF-1 were highly expressed in hypoxic HepG2 cells compared with the normoxia control group. FITC, fluorescein isothiocyanate; HIF, hypoxia inducible factor; TRITC, tetramethylrhodamine isothiocyanate; IGF, insulin-like growth factor.

To detect the expression of HIF-1α or IGF-1 on the membrane of hypoxic HepG2 cells, an immunofluorescence staining assay was performed. The results revealed that HIF-1α and IGF-1 were highly expressed in hypoxic HepG2 cells compared with the normoxia control group (Fig. 4).

### Effect of CoCl_2_-induced hypoxia on the production of VEGF

**Figure 5:**
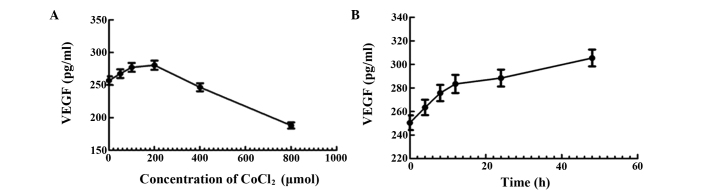
Effect of CoCl_2_-induced hypoxia on the production of VEGF was determined by ELISA. (A) HepG2 cells were treated with 0, 50, 100, 200, 400 or 800 μmol/l of CoCl_2_ for 12 h. (B) HepG2 cells were treated for 0, 4, 8, 12, 24 or 48 h with 200 μmol/l CoCl_2_. VEGF, vascular endothelial growth factor; CoCl_2_, cobalt chloride.

The effect of various concentrations and durations of CoCl_2_ treatment on the production of VEGF in HepG2 cells was assessed using an ELISA assay. The results revealed that the production of VEGF increased with increased concentration and time, and it reached a peak at a concentration of 200 μmol/l CoCl_2_ (Fig. 5A and B).

## Discussion

Hypoxic microenvironments and angiogenesis have been a focus of tumor research over previous years. The expression of numerous genes has been found to be regulated by hypoxic microenvironments, which plays a crucial role in biological characteristics. In the present study, CoCl_2_ was utilized to mimic hypoxia. Ferrochelatase is inactived by a substitution performed by cobalt ions under normoxic conditions, which blocks oxygen absorptivity in cytoblasts. A hypoxic microenvironment is subsequently formed (11).

Maintaining the oxygen balance is key for cell survival. A series of abilities have evolved that adapt to the changing oxygen concentrations over the process of development. If the proliferation of tumor cells reaches or exceeds the speed of angiogenesis, a hypoxic microenvironment is formed. HIF-1 is a transcription factor found in mammalian cells cultured under reduced oxygen tension, and plays an essential role in cellular and systemic homeostatic responses to hypoxic microenvironments, including the regulation of genes involved in energy metabolism, angiogenesis and apoptosis. HIF-1α is rapidly degraded by the proteasome under normal conditions but is stabilized by hypoxic conditions (12). The process of angiogenesis possesses multiple steps, which is fundamental for tumor growth, invasion and metastasis. Growth factors are over-expressed and released, resulting in tumor over-growth. This promotes the vascular endothelial cells to migrate from the host vessel to tumor tissue. HIF-1α and IGF-1 stimulate vascular endothelial cell migration, proliferation, differentiation and angiogenesis (13).

The present study identified that the cell viability worsened with increasing concentrations of CoCl_2_, which may be correlated with the cytotoxicity of CoCl_2_. Furthermore, it was found that the HIF-1α, IGF-1R and VEGF proteins were positively correlated with CoCl_2_, and therefore hypoxia, in a dose- and time-dependent manner, demonstrated by the various concentrations and incubation times. This aided investigation into the best hypoxic conditions for solid tumor studies.

Previous studies have revealed that cell death, protein oxygenization and lipid preoxidation decrease when the expression of the HIF-1α and IGF-1 proteins is normal, which promotes the formation of novel blood vessels and the proliferation of cells, inhibiting apoptosis. If not, the levels of lactate dehydrogenase increase and the level of glutathione S-transferases decreases, resulting in the inhibition of the formation of novel blood vessels, which reduces the function of the cell membrane (14,15). This is one possible explanation for the small change or decrease in mRNA and protein levels compared with the control group when the concentration reached 800 μmol/l or the incubation time was ≥24 h. VEGF production declined in HepG2 cells once the concentration of CoCl_2_ was >200 μmol/l. In addition, the side-effects of the increased drug concentration on the cells must be considered.

IGF-1 is a mitogenic and anti-apoptotic growth factor that regulates cellular proliferation, differentiation and cell death. The effects of IGF-1 are mediated through growth hormones, oncogenes, anti-oncogenes and hypoxic microenvironments (16). Previous studies have found that abnormal levels of IGF-1 in malignant tumors, including breast, lung, prostate and liver cancers, may be correlated with autocrine IGF-1 expression (17–20). In the present study, it was found that HepG2 cells in hypoxic conditions can secrete IGF-1, which was positively correlated with CoCl_2_, and therefore hypoxia, in a dose- and time-dependent manner. The biological activities of IGF-1 are mediated via the IGF-1 receptor (IGF-1R), which belongs to the receptor tyrosine kinase family of membrane receptors. The effects of IGF-1R on cell growth and apoptosis are mediated through binding to IGF-binding proteins (IGFBPs) in the circulation. Following receptor and IGFBP activation, two canonical signaling cascades are activated, the phospho-inositide-3-kinase (PI3K) and mitogen-activated protein kinase (MAPK) pathways. Ultimately, the activation of the PI3K and MAPK pathways governs their specific effects on cellular behavior. When adaptor molecules, including insulin receptor substrates, are recruited and undergo tyrosine phosphorylation, the PI3K pathway is activated. Subsequently, there is an increase in the levels of the membrane-bound phospholipid phosphatidyl inositol-3,4,5-triphosphate and the recruitment of Akt to the membrane. Akt is a central mediator of the intracellular effects of IGF-1R and is involved in metabolism, cell survival, cell migration and proliferation through various mechanisms. Activation of the MAPK pathway by IGF-1 also occurs downstream of adaptor proteins, including MAPK kinase kinase, Raf, MAPK kinase, MEK and MAPK. Extracellular-signal-regulated kinase 1/2 then exerts mitogenic and inflammatory effects (21,22). Previous studies have found that activation of the PI3K and MAPK pathway can induce the activation of the VEGF receptor pathway that is mediated by IGF-1, through the regulation of HIF-1α (23–26). It has also been reported that the IGF system suppresses angiogenesis through the PI3K/HIF-1α/VEGF signaling pathways in a hypoxic microenvironment in ovarian cancer (27). This is consistent with the findings of the present study, but additional investigation is required to determine whether the decrease in VEGF is due to the PI3K/HIF-1α signaling pathway.

In summary, the present study has demonstrated that CoCl_2_ can induce HepG2 cells under hypoxic conditions at a moderate concentration and appropriate incubation time. The results presented in the current study indicated that IGF-1, which is secreted by hypoxic HepG2 cells, can promote the accumulation of HIF-1α mRNA and protein. Subsequently, HIF-1α regulated the expression of VEGF mRNA and protein in hypoxic HepG2 cells. VEGF is closely associated with the development and metastasis of HCC, and inhibition of IGF-1 or HIF-1α may be an promising target for hepatocellular carcinoma. However, whether certain other underlying molecular mechanisms promote the accumulation of HIF-1α to regulate VEGF expression remains poorly understood.

## Figures and Tables

**Table I:** The correlation in the does dependence between hypoxia induced and gene expression.

	CoCl_2_, μmol/l					
	
Protein	0	50	100	200	400	800
HIF-1α	0.41+0.10	0.65+0.06	0.85+0.04	0.88+0.04^a^	1.33+0.05^a^	0.78+0.08
IGF-1R	0.06±0.01	0.15±0.06	0.18±0.03	0.29±0.08^a^	0.38±0.11^a^	0.13±0.08
VEGF	0.87±0.08	1.48±0.85	1.82±0.67	2.33±0.85^a^	2.98±1.08^a^	1.76±0.19

a P<0.05 compared with control group (0 μmol/l).

CoCl_2_, cobalt chloride; HIF-1α, hypoxia inducible factor-1α; ICF-1R, insulin-like growth factor-1 receptor; VEGF, vascular endothelial growth factor.

**Table II:** The correlation in the time dependence between hypoxia induced and gene expression.

	Time, h					
	
Protein	0	4	8	12	24	48
HIF-1α	0.36+0.06	0.42+0.05	0.72+0.03	0.87+0.03^a^	1.23+0.05^a^	1.18+0.02^a^
IGF-1R	0.22±0.02	0.25±0.03	0.27±0.03	0.67±0.12^a^	0.65±0.17^a^	0.78±0.15^a^
VEGF	0.43±0.05	0.48±0.07	0.55±0.08	1.85±0.75^a^	2.39±0.98^a^	2.56±0.89^a^

a P<0.05 compared with control group (0 μmol/l).

HIF-1α, hypoxia inducible factor-1α; ICF-1R, insulin-like growth factor-1 receptor; VEGF, vascular endothelial growth factor.

**Table III:** Dose-dependent correlation between hypoxia and gene expression.

	CoCl_2_, μmol/l					
	
Gene	0	50	100	200	400	800
HIF-1α	0.56±0.06	0.78±0.03	0.88±0.23	1.47±0.4^a^	1.53±0.58^a^	1.18±0.22
IGF-1R	0.64±0.07	0.81±0.11	1.01±0.43	1.46±0.3^a^	1.51±0.67^a^	0.87±0.08
VEGF	0.71±0.08	1.03±0.15	1.14±0.16	1.62±0.7^a^	1.66±0.83^a^	0.97±0.09

a P<0.05 compared with the control group (0 μmol/l).

CoCl_2_, cobalt chloride; HIF-1α, hypoxia inducible factor-1α; ICF-1R, insulin-like growth factor-1 receptor; VEGF, vascular endothelial growth factor.

**Table IV:** Time-dependent correlation between hypoxia and gene expression.

	Time, h					
	
Gene	0	4	8	12	24	48
HIF-1α	0.45±0.07	0.55±0.08	1.30±0.12^a^	1.63±0.1^a^	1.73±0.11^a^	2.14±0.2^a^
IGF-1R	0.31±0.03	0.40±0.10	1.10±0.10^a^	1.51±0.0^a^	1.87±0.11^a^	2.68±0.8^a^
VEGF	0.65±0.05	0.85±0.02	2.14±0.58^a^	2.37±0.6^a^	2.53±0.58^a^	3.10±0.6^a^

a P<0.05 compared with the control group (0 μmol/l).

HIF-1α, hypoxia inducible factor-1α; ICF-1R, insulin-like growth factor-1 receptor; VEGF, vascular endothelial growth factor.
